# Embolization of an Atrial Septal Defect Occluder Device Into the Left Ventricle

**DOI:** 10.7759/cureus.11417

**Published:** 2020-11-10

**Authors:** Bryan J Hierlmeier, Galina Ostrovsky, Matthew Zarth

**Affiliations:** 1 Anesthesiology, University of Mississippi Medical Center, Jackson, USA

**Keywords:** atrial septal defect (asd), amplatzer, embolization, 3d-echo, echocardiography, abandoned device, malposition

## Abstract

Amplatzer Atrial Septal Occluder device has been routinely and successfully used as a percutaneous alternative to cardiac surgery for closure of atrial septal defects. It has shown to the safe with a low complication profile. Complications that most commonly occur with atrial septal defect (ASD) closure devices include malposition or embolization, residual shunt, atrial arrhythmias, thrombosis over the vena cava or atrium, erosion and perforation of the heart, and infective endocarditis. The most common complications associated with an ASD occluder device appear to be embolization and malposition with embolization usually occurring in the main pulmonary artery.^ ^We present a case in which the ASO device, Amplatzer^TM^ (Abbott, USA), embolized into the left ventricle.

## Introduction

Atrial septal defect (ASD) is one of the most common congenital cardiac malformations [1-3]. ASD transcatheter occlusion techniques can use various devices, including the Amplatzer Septal Occluder, and have become the alternative to the surgical treatment of ASD [1-4]. However, complications using such devices have been reported, including embolization, malposition, erosion, arrhythmias, endocarditis, and thromboembolic events [1,4-6]. We present a patient with a large ASD who had an episode of emesis following an Amplatzer^TM^ (Abbott, USA) closure device placement, which likely led to embolization of the device across the atrial septum and into the left ventricle.

## Case presentation

**Figure 1:**
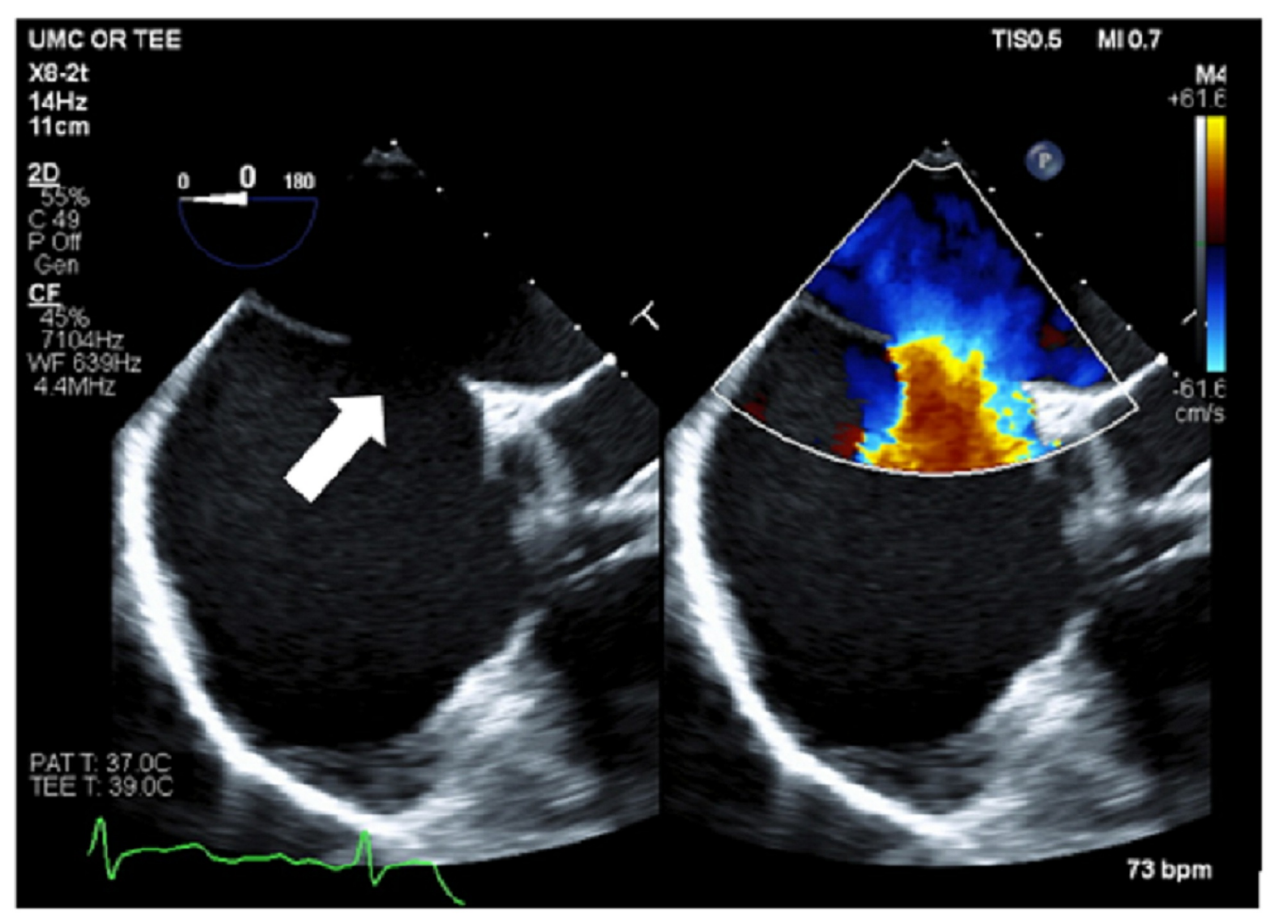
Mid-esophageal 4 chamber view showing the large ASD (white arrow) with left to right shunting using color flow Doppler. ASD: atrial septal defect

A 55-year-old female with a past medical history of hypertension initially presented to the hospital with shortness of breath and found to be in a new-onset atrial flutter with 2:1 block and a ventricular rate of 140 beats per minute. She was admitted for rate control and underwent a transesophageal echo (TEE) to evaluate a pansystolic 3/6 murmur best heard over the third intercostal space on the left. During the TEE evaluation, she was noted to have a large secundum atrial septal defect measuring 2.0 cm x 2.4 cm, with a left to right shunt (Figure 1) and severe right ventricle dilation with normal biventricular function. 

**Figure 2:**
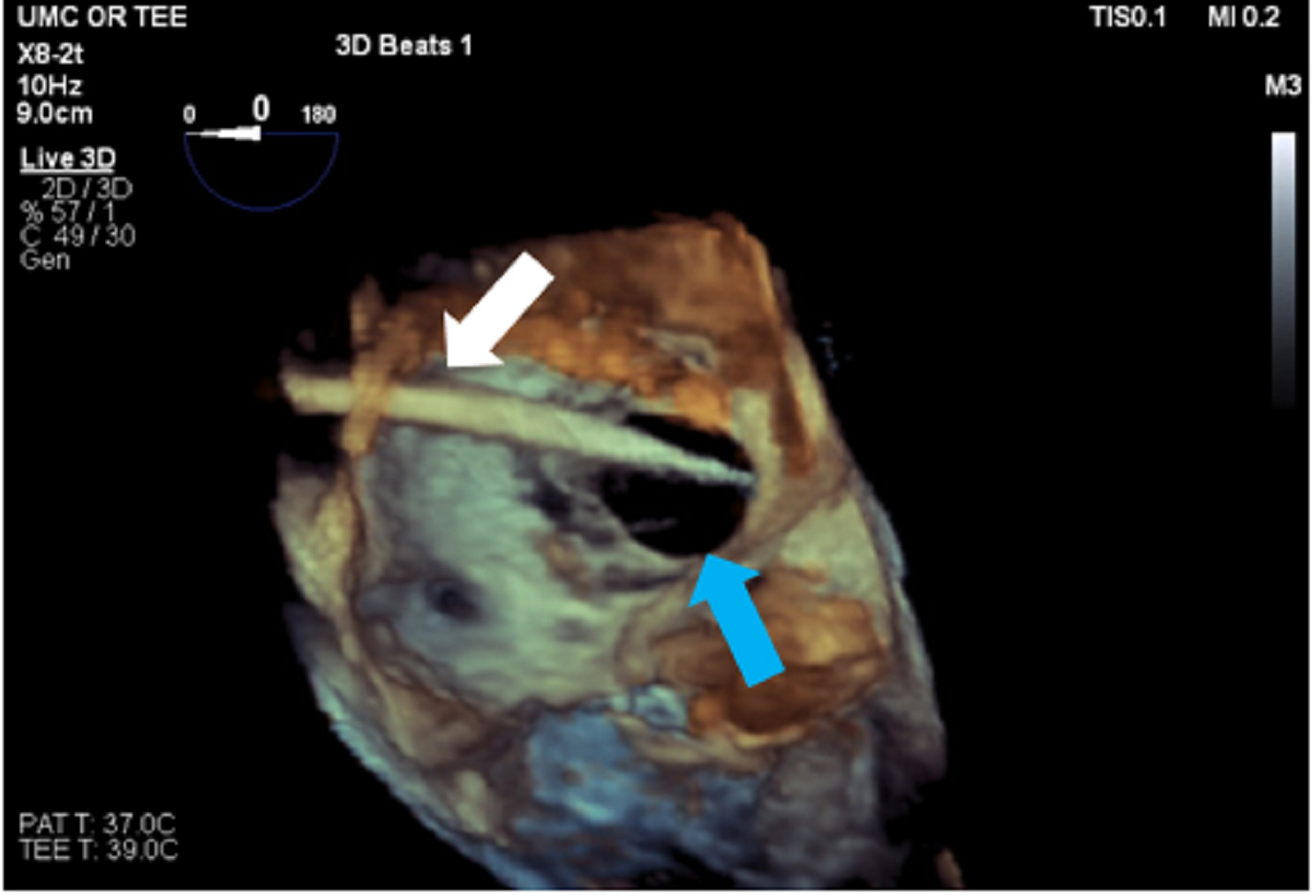
Three-dimensional view from right atrium showing the deployment catheter (white arrow) going through the ASD (blue arrow) into the left atrium. ASD: atrial septal defect

She was evaluated by the structural heart team and later posted for ASD closure in the interventional suite. Later, she was brought to the interventional suite and standard American Society of Anesthesiologists monitors (ASA) were applied. Anesthesia induction was completed with propofol, fentanyl, and rocuronium, and the patient was intubated uneventfully. The insertion of the TEE probe confirmed the large ASD with a left to right shunt as seen in Figure 1. Vascular access was obtained by the Interventional Cardiologist. A 6F catheter was advanced over a wire into the right atrium and across the ASD as seen in Figure 2.

**Figure 3:**
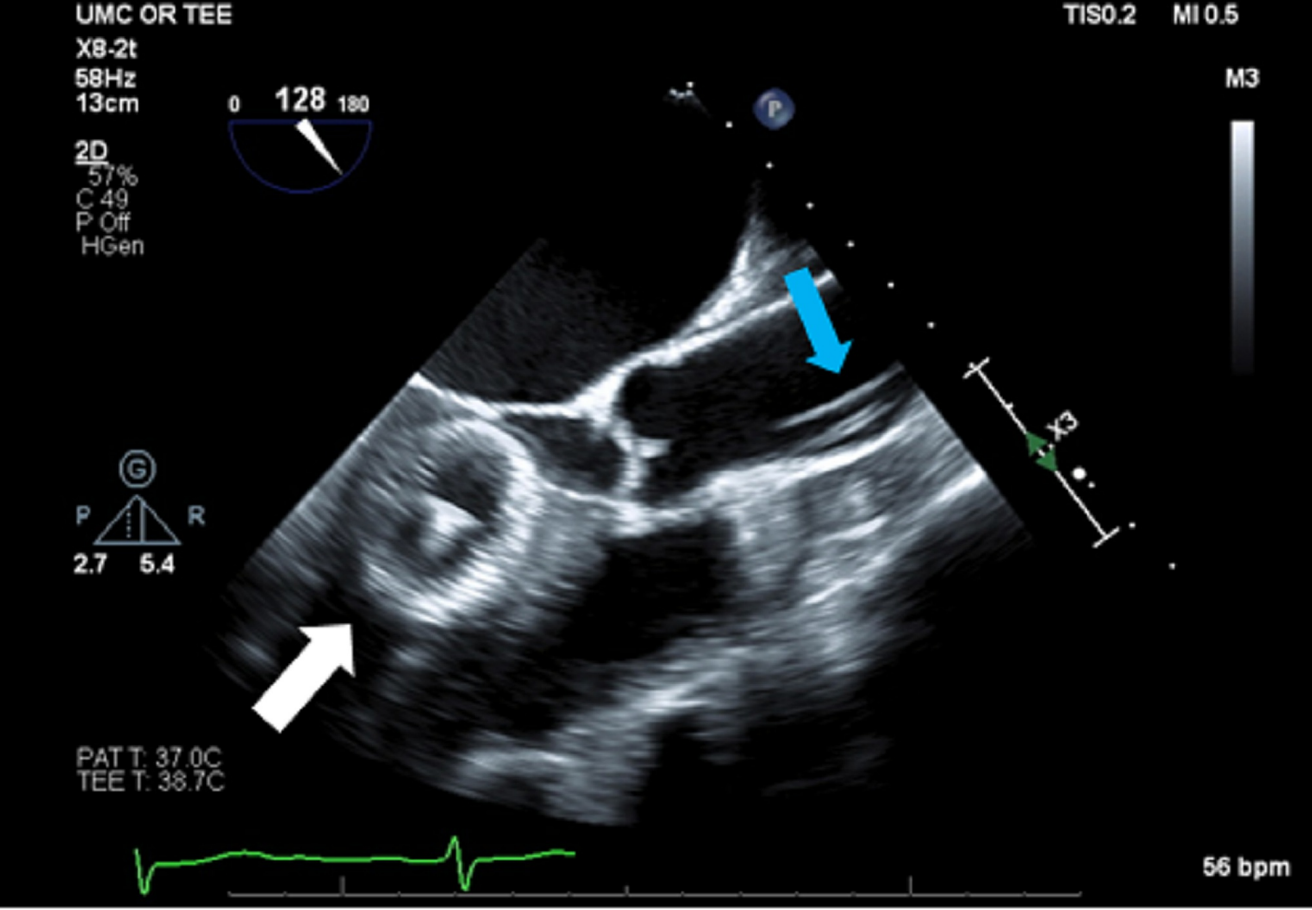
Mid-esophageal long axis view showing the Amplatzer device (white arrow) in the left ventricle with near occlusion of the LVOT and the retrieval catheter (blue arrow) in the ascending aorta. LVOT: left ventricular outflow tract

**Figure 4:**
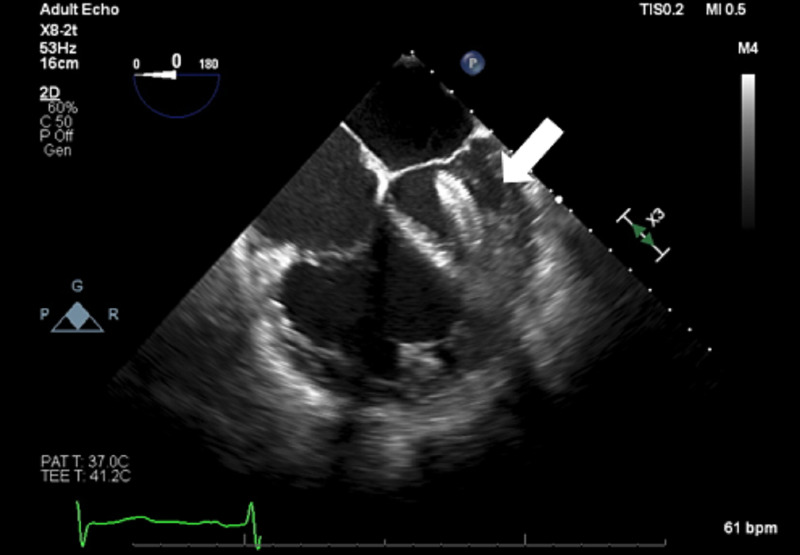
Mid-esophageal 4 chamber view showing a dilated right ventricle and the device in the left ventricle (white arrow).

Balloon sizing was done and a 24 mm Amplatzer Atrial Septal Occluder device was inserted across the atrial septum showing a good seal with no residual flow across the septal margins on TEE. After the procedure, the patient was extubated and taken to the recovery room before being transferred to the floor. Transthoracic echo the following day showed that the occluder device had embolized into the left ventricle. The patient had been hemodynamically stable all night but did have two episodes of emesis followed by a few episodes of ectopy on her electrocardiogram. The patient was taken back to the interventional suite for retrieval of the device from the left ventricle. TEE images of the device in the left ventricle are seen in Figures 3, 4.

**Figure 5:**
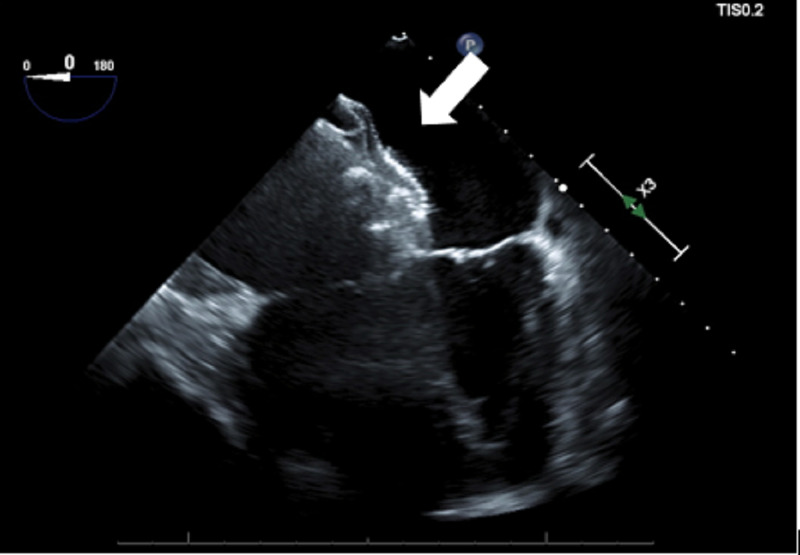
Mid-esophageal 4 chamber view with proper deployment of the Amplatzer closure device (white arrow).

The device was retrieved by introducing a sheath into the femoral artery and crossing the aortic valve to snare the 24 mm device. This was completed without incident leaving the aortic valve intact. A 28 mm device was successfully placed across the atrial septum using femoral vein access leaving no residual flow across the atrial septum on intra-op TEE as seen in Figure 5.

Transthoracic echo on the day following the procedure showed that the device was still in place across the atrial septum with no residual leak.

## Discussion

Amplatzer Atrial Septal Occluder device has been routinely and successfully used as a percutaneous alternative to conventional cardiac surgery for closure of atrial septal defects. Transcatheter closure of secundum type of ASD has become a standard treatment that is less invasive and presents with fewer complications and shorter recovery time than surgery, which requires cardiopulmonary bypass [1-6]. The occlusion techniques using this device include classic percutaneous ASD occlusion under fluoroscopy, percutaneous procedure under ultrasound-guidance, and intra-operative device close under ultrasound-guidance [1]. While percutaneous occlusion is generally considered effective and safe for patients with secundum ASD, limitations with this technique using fluoroscopy include damaging radiation exposure and higher risk for complications for children under 10 years of age [1,2]. The size and rim of the ASD in addition to the size of the occluder are also important to consider when using the percutaneous approach.

The transcatheter closure technique for treating ASDs is favored over surgery due to its minimal invasiveness, lower morbidity, lower mortality, and shorter hospital stay and recovery time [1-6]. However, there are some risks and complications of note that may arise upon placement of an ASD occluder device. Complications associated with ASD closure devices include malposition or embolization, residual shunt, atrial arrhythmias, thrombosis over the vena cava or atrium, erosion and perforation of the heart, and infective endocarditis [1,4,6]. The incidence of device embolization is reported to be about 0.5% in experienced hands, with successful percutaneous retrieval being reported in approximately 70% of the cases [7]. A large ASD, thin atrial rim, incorrect placement of the device, and under or oversizing of the device are also risk factors for embolization [5]. If embolization occurs, a gooseneck snare or basket catheter is used to retrieve the device, or in cases using larger size devices, the patient may undergo surgery to retrieve the device and close the ASD [4]. In cases where malposition of the device occurred, the device is replaced with a larger diameter device [4].

There are several different devices available for transcatheter ASD closure, each with different advantages and disadvantages. The Amplatzer Septal Occluder (ASO) has been the preferred device for closing defects larger than 18 mm [4]. To avoid accidental detachment of the ASO, the cable needs to be screwed on the device again to prevent accidental detachment, which would make retrieving the ASO very difficult. In addition to malposition, device erosion has been reported to be a long-term complication with the Amplatzer Septal Occluder, as oversizing (1.5 times the size of the ASD) increases the risk of erosion [6]. Our case is unique as the ASO ended up in the left ventricle instead of the more common right ventricle or pulmonary artery due to the left to right atrial shunting. We believe that during the patient’s episodes of emesis on the night of her first procedure that her right atrial pressure increased to an extent that dislodged the device across the septum and into the left atrium followed by the left ventricle.

## Conclusions

Atrial septal defect is one of the most common congenital heart defects, and transcatheter occlusion of ASD using an Amplatzer closure device has been shown to be safe and effective with a low rate of complications. This approach is favored over surgery as it is less invasive and poses less serious risks. Understanding the risks with percutaneous closure is important in reducing potential complications. It is also important to consider the size and anatomy of the defect as well as the size of the device and to confirm proper seating of device anchors with septal rims, this avoids later embolization. Dislodgement and embolization of the device more commonly end up in the right ventricle or pulmonary artery due to left to right shunting but may end up in the left atrium or left ventricle if the shunt is reversed. 
